# Forensic exploitation of patterned injuries: Promoting structured analysis as an early assessment for comparison process

**DOI:** 10.1016/j.fsisyn.2024.100469

**Published:** 2024-04-23

**Authors:** Stella Fahrni, Olivier Delémont, Silke Grabherr

**Affiliations:** a School of Criminal Justice, University of Lausanne, 1015, Lausanne-Dorigny, Switzerland; b University Centre for Legal Medicine Lausanne-Geneva, Chemin de la Vulliette 4, 1000 Lausanne 25, Switzerland

**Keywords:** Forensic imaging, Photography, 3D surface scanning, Patterned injury, Lesion

## Abstract

Practice at our Center shows that approach using 3D surface imaging for morphometric comparison of patterned injuries does not always lead to accurate conclusions.

We decided to evaluate whether a selection protocol focused on analysis phase could enable us to form an early assessment of the outcome of a comparison process, and then to select lesions likely to lead to a probative conclusion.

23 blunt objects were used to create 65 patterned injuries on an experimental model simulating human skin. A blinded analysis and a comparison were conducted on photographs and 3D models of the lesions. Statement of analysis phase was consistent with comparison results in most cases, enabling correct identification of the responsible object or at least keeping it as possibly responsible among 2 to 3 objects.

Our protocol has been demonstrated to improve ability to exploit patterned injuries from surface imaging, despite certain limiting factors.

## 1 Introduction

Since February 2014, numerous cases have been treated in our legal medicine centre using 3D imaging techniques. In a large proportion of these cases, a morphometric comparison between a particular type of lesion and suspect blunt object(s) was undertaken [1]. These particular lesions are called “patterned injuries” [2,3] because they correspond to lesions with specific shapes and physical characteristics, which are linked to a specific object or action and can therefore be compared with the object. This type of lesion is of particular interest in forensic medicine when reconstructing the course of an accident or a crime [[4], [5], [6], [7], [8], [9], [10], [11], [12]]. Most of the time, blunt objects, shoe soles, tires, etc. cause this type of lesion. The study of these cases and the more general analysis of the 3D imaging approach that we follow at our centre have highlighted the limits of this technological contribution, its methodological shortcomings, and their consequences on the conclusion given in reports and to the prosecutor. Indeed, when a suspicious object is confiscated by the police, a superposition between its 3D model and that of the lesion is made directly. However, in many situations, comparisons by superimpositions between the 3D models of the lesion and the suspect object did not make it possible to pronounce on the possibility that the said object has created this lesion. This is due to the fact that the lesions considered often have few visible elements, are blurred and are therefore not very informative. Moreover, deformations are considered, case by case according to the operator's estimation but without any real rule. Sometimes, in the case of an imperfect match, we conclude that the object may have created the trace, sometimes not. Thus, the conclusions provided are often not very useful in relation to the time and money invested.

Examining the pertaining literature, we observe an over-representation of case reports, yet convincing, and only a very small number of systematic and rigorous basic research studies [[13], [14], [15]]. Even in these rigorous studies, there is no clear statement of the criteria used to associate a lesion with a suspected blunt object. In one of these studies, high wrong correlation rate between a lesion and the injury causing instrument was found applying some basic criteria; thus, they could conclude that “*analysis phase appears as an imperative step before any comparison in order to prevent misinterpretations, false positives or over-determination in conclusion.”* In another study, the authors mention subjective experimental comparisons of 3D data on forensic skin as well as qualitative and quantitative assessments without giving many details on the criteria but conclude that the results obtained are statistically significant for assessing the quality of the scanned surface. For the time being, and to our knowledge, the results of such lesion-object comparisons do not seem to have been challenged in court. However, we believe that this may change in the future, and we believe that it is essential to be able to base results and conclusions on a systematic, transparent and rigorous method of comparison.

Therefore, the aim of our research is to set up an experimental study to assess if the development of an approach based on ACE-V (Analysis Comparison Evaluation - Verification) could be beneficial for the exploitation of patterned injuries applying 3D surface imaging (3DSS); and then to lay the foundations for such a method. This approach has proven its usefulness and reliability in forensic science for the exploitation of various traces (fingermarks, shoe marks, toolmarks, etc.). A lesion can be considered as a trace: it is the vestige of a past action and just like a fingermark or a shoemark, it can be exploited to determine its source (what created it) or to explain the course of an event (how it was created) [[16], [17], [18], [19], [20], [21], [22], [23]]. Our approach emphasises the analysis phase that is used to measure the potential and the limits of the information conveyed from the lesion by describing and evaluating different features of the lesion related to its pattern (general, quality, characteristics, etc) and the deformations and rotations undergone during its production [[24], [25], [26]]. This step should make it possible to clarify the criteria to be recorded on a lesion. These criteria should serve as a basis to

1. Determine what degree of association can be expected;
2. Guide the comparison phase.

The various criteria recorded on the lesion make it possible to estimate its informative potential and thus to know to what degree this lesion could be exploited. This makes it possible to define whether a comparison would be relevant or useless. If a comparison is possible, it makes it possible to determine which elements should be sought on the reference lesion, which measures to take, and it provides a more solid basis for justifying the conclusions which the examiner will reach. Thus the results obtained from these experiments can be used to develop a working method for this type of lesion.

## 2 Material and methods

**Fig. 1:**
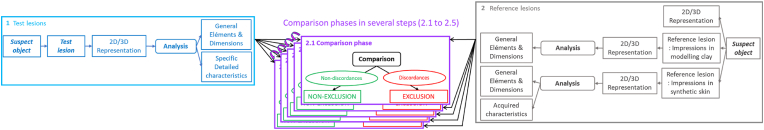
Resume of process followed during the study.

This study was divided in sequential steps: a) choice of material and production of test lesions, b) 2D and 3D recording of test lesions, c) analysis phase for each type of representation of each lesion, d) comparison of test lesions with reference lesions. A schematic view of this experimental process is presented in Fig. 1 and the different steps are explained in this section.

### 2.1 Preparation of test and reference lesions

**Fig. 2:**
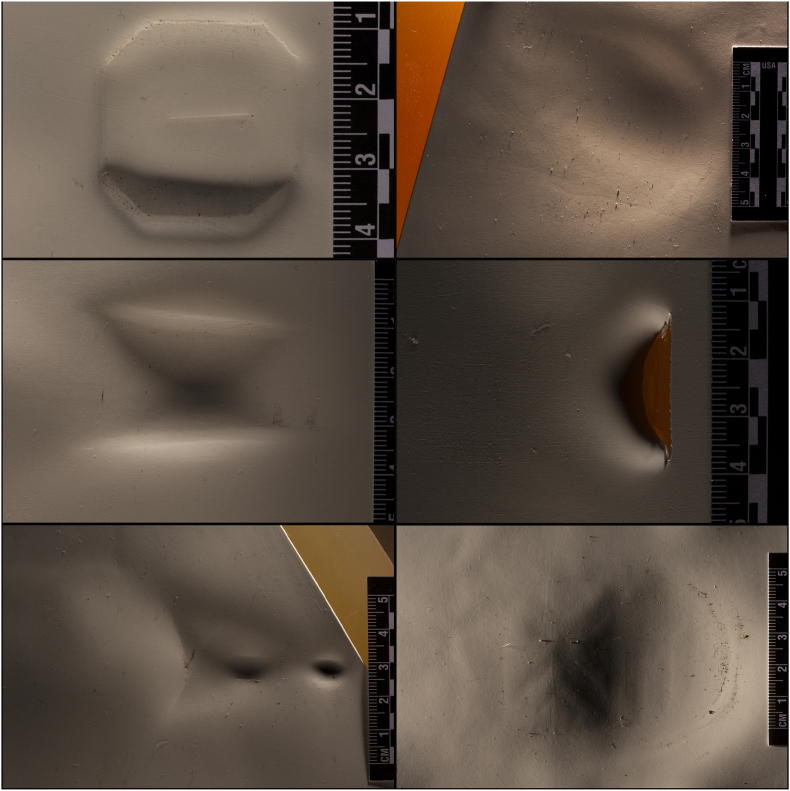
2D documentation of a selection of test lesions.

65 simulation injuries referred as “test lesions” (named L001 to L060, L111 to L115) were produced by volunteers on a particular assembly using different blunt tools: hammers, crowbars, golf clubs, shovels, staffs and shoe soles (Step 1 on Figs. 1 and 2). Three examples of each type of blunt object and 4 pairs of shoes were used. This selection of 23 panel objects was made based on the blunt objects mostly used in cases treated in our institute since 2008. A sheet of synthetic (silicone) skin (20,3 × 15,2 × 0,1 cm) (ordered on Amazon with reference name Mlmsy Tattoo 8 × 6″ Silicon Matériel peau pratiques de tatouage pour les débutants et artistes expérimentés”, manufacturer: MLMSY, ASIN: B01LYZE4O7, manufacturer reference: 95871246385), fixed on a bloc of ballistic soap (25 × 25 × 4 cm) (Mettler AG), composed each assembly, in order to simulate human skin and sub-cutaneous tissues respectively. This way of producing lesion was chosen because it results in a realistic blunt force trauma pattern [14,[27], [28], [29]]. Each lesion as well as each tool was individually labelled. The four volunteers who hit the assembly were 2 women and 2 men aged between 29 and 53 years. No specific instructions were given to them, just to strike the assembly as if they would like to hurt somebody. There were also no instructions on the number of injuries made per type of tool. Volunteers then filled in a form giving all details related to the tool used and conditions of lesion production. This phase took place indoors. The main researcher in charge of the study did not take part to this production phase of test lesions, nor was she informed about which tool was used to produce which lesion. This way of processing allowed us to conduct a blind study.

For comparison needs, “reference lesions” were created by the main researcher (29 years old woman) in a favourable material (modelling clay) and in a similar material (synthetic skin) to that of the test lesions with the panel of objects (Step 2 on Fig. 1). The favourable material allows the most complete reproduction of the pattern and features present on the object used, while the similar material makes it possible to obtain an impression similar to that observed on the test lesion if the same object caused it, (thus often not exhibiting all the features of the object). This should allow for a more reliable comparison. One low-intensity and one high-intensity blow were inflicted in each material in order to cover a range of blow intensity for each test. Sometimes several different configurations i.e., different angles of blows or different face of the object impressed in the material of the object were used.

### 2.2 2D documentation

Each lesion was photographed following a recording procedure developed specifically for this study inspired by our experience and literature [[30], [31], [32], [33], [34], [35]] (“2D/3D Representation” in Step 1 on Fig. 1). A Canon EOS 6D camera, 100 mm macro lens. ISO: 100, exposure time 1/60, white balance flash was used. The aperture was adjusted accordingly, and the sharpness was set manually. These photographs were taken on a fixed reproduction bench, with a dimension marker and lighting parallel to the surface of the trace, in order to ensure a high degree of standardization.

### 2.3 3D scanning

Two 3D surface scanners were used for the experiments: the GOM ATOS COMPACT SCAN 5 M (GOM, Braunschweig, Germany) and the CREAFORM Go!SCAN 3D (CREAFORM, Canada). These instruments are used routinely in our institute for 3D surface acquisition of lesions, bodies and objects. The first one allows the best resolution but does not provide a 3D colour model [27,36,37]. The details potentially present on the lesions can be very small (less than a millimetre). Thus, the best resolution possible is desirable while maintaining the ability to scan the entire lesion within an acceptable time.

The CREAFORM Go!SCAN 3D scanner makes it possible to obtain a 3D model in colour, but at a lower resolution than with the GOM scanner. Based on our experience, a suitable measurement volume (MV) has therefore been chosen for each scanner.

The scanning parameters (GOM: min fringe contrast 10, use of points at strong brightness differences, use of points on shiny surfaces, avoid Mx viewing angle sensor/surface, exposition time: 2; Go!SCAN: resolution 1 mm) for each apparatus were set based on our experience in previous research [14,27] and during routine work at our institute.

3D surface measurement was first processed through the non-contact optical 3D digitising system GOM ATOS Compact Scan 5 M, which allows to obtain 3D models from real data with high resolution and accuracy. The functioning of this scanner relies on the fringe pattern projection (blue light) of adapting an array of stripes that impact the surface to be measured. Two 5-million-pixels cameras record the deflections of the stripes induced on the shape of the surface. By triangulation principle, the measurements from both cameras are merged in a point cloud representing the surface, with high resolution and accuracy [36,37]. Acquisition was controlled through the ATOS Professional V7.5 SR2 software package, also used for some further treatments in conjunction with a 3ds Max 2013 software. A MV – corresponding to a pair of camera lenses – was used on the scanner: MV150 (150 × 110 × 110 mm) which has a resolution up to 0.062 mm (“2D/3D Representation” in Step 1 on Fig. 1). A complete operation of 3D data acquisition for a lesion required an average of 15–30 min.

A calibration of the scanner was carried out before each session of scanning, to assure minimal deviation in measurements. Markers were placed around the lesions which were scanned on a black turntable in a room at ambient temperature (around 21 °C) under controlled and stable luminosity. Parasite movements were reduced to a minimum level by fixing the different parts of the camera to a tripod and by working in an isolated room.

Data were then polygonised without post processing. The only other treatments applied to the obtained models were smoothing and thinning to remove existing background noise and reducing 3D model size by simplifying information in relation to the curvatures of the zones.

3D surface measurement was processed through a second non-contact optical 3D digitising system called CREAFORM Go!SCAN 3D. This device also works according to the triangulation principle. However, it does not produce blue light, but white light and the device is equipped with an additional colour camera. Thus, both 3D and texture data can be captured during the scan. The scanned object appears in real time in the VxElements software [38]. Once the scan is complete, the raw data are polygonised to obtain the final model. The highest resolution that can be achieved is 0.1 mm with the Go!SCAN 20 and 0.5 mm with the Go!SCAN 50. The higher the required resolution, the slower was the data acquisition.

Each lesion was scanned with the Go!SCAN 20, set up with the standard resolution of 1 mm (“2D/3D Representation” in Step 1 on Fig. 1). Specific targets for this scan were placed around the lesion. Scans occurred in same conditions as for GOM.

3D models obtained were then cleaned and reduced in the VxElements software as described above.

The reference lesions were recorded using the same three techniques, as described above (“2D/3D Representation” in Step 2 on Fig. 1).

### 2.4 Analysis phase

The 2D (65), 3D (65) and 3D colour (65) representations (Total of 195) of the test lesions were examined (Step 1 on Fig. 1). An electronic form was used as a canvas to collect information on all traces for this phase of analysis. The form had five sections on the lesion: general information, clarity and quality, dimensions, observed features and conclusion. General information was used to reference the analysed lesion with its type, the type of object that could have created it, as well as the substrate in which it was created, and the type of technique used to register the analysed lesion. According to Ashbaugh [24], “How well the details from 3-D ridges that are reproduced in the 2-D print is referred to as the clarity of the print”, we applied this concept used originally for fingerprint examination to our lesion examination. The clarity of the trace takes into consideration any visible signs of distortion, the accentuation of the marking of the lesion and what is observable to determine the general quality of the lesion and how to subsequently estimate/assess the visible features. More explanations about the concept of clarity are available in the articles of Ashbaugh [24] and of Langenburg and Champod [24,25]. The dimensions are the visible maximal length and width of the lesion. Sometimes, certain limits of the lesion are not noticeable, because the measurement stops at the last visible point. The observed features (referred as General Elements on Fig. 1) start with general features of the lesion such as its shape, general pattern, colour or other inscription, and then continue with specific detailed characteristics. A coding system for the characteristics of the traces according to their clarity developed by Langenburg and Champod [25] was applied. This system makes it possible to represent by a colour the confidence attributed to the existence of the observed characteristic: Green for good confidence, Yellow for medium confidence and Red for low confidence. Finally, the conclusion of the lesion's analysis is formulated and argued according to the observations made during the analysis phase:

- Unexploitable: The lesion has no or few measurable characteristics that are not sufficient in quantity and quality to allow a comparison process to be carried out and to lead to an exclusion/non-exclusion decision. There is therefore no need to continue the process.
- Exploitable: The lesion has sufficient measurable characteristics in terms of quantity and quality to allow a comparison to be made, leading to at least the conclusion of exclusion/non-exclusion.

The quality of the lesion and the conclusion of the analysis are the two main elements of the analysis of a lesion. The overall quality of the lesion has a strong impact on the decision taken at the time of the conclusion even if additional elements also influence it.

To analyse the photographs of lesions, the “Image J” software (NSI, USA) was chosen; and to analyse the 3D models of lesions, the Meshlab software (ISTI & CNR, Italy) was chosen. Choices of software were made based on the possibility to draw on the lesion, the easiness of handling for the user and its free access. To enable the reader to better understand the analysis process, the analysis of the L030 lesion by the bias of its photograph is shown in Supplementary Materials. The image file has been opened in the Image J software and the measuring range has been set by means of the green line on the scale. The elements visible on the lesion were marked in green, when high confidence in what was observed was achieved and yellow when confidence was medium.

Reference lesions were also analysed following a similar protocol to the one used on the test lesions (Step 2 on Fig. 1), with just a few more analysed elements. The variability of the impressions produced by the object was estimated on the modelling clay by looking at the strong and weak impressions (thanks to strong and weak blows) and noting their dimensions and general characteristics. The same procedure was undertaken for reference lesions on the synthetic skin. Finally, the acquired characteristics present on the reference lesions created in the synthetic skin were recorded. Randomly acquired characteristics referred to features resulting from the use of a known object or the wearing of known shoes. These characteristics can be used to restrict the field of tools that could have produced this lesion (reduction factor), in the extreme case to the point of allowing the lesion to be individualized (highest level of non-exclusion).

### 2.5 Comparison phase

The comparison phase in this research allowed the conclusions of the analysis phase to be validated. Thus, 12 test lesions with a wide range of general shapes and detail features deemed exploitable at the end of the analysis were selected for comparison. As full dataset was too big, this number of 12 lesions was selected to obtain a data size manageable for downstream analysis.

The comparison was then carried out in five phases for each test lesion with the reference lesions through each representation (2D, 3D and 3D colour) (Phase 2.1 to 2.5 on Fig. 1). Phase 2.1 was to compare the test lesion and the reference lesions created in the modelling clay of each selected object based on the general elements and dimensions and then to exclude the reference lesions for which discordances (different shape of the lesion, divergent borders, absence of a pattern seen in the lesion, dimensions too far apart) were observed based on these two criteria. Because no impression is ever perfectly replicated, comparative measurements must be within acceptable tolerance for variations [26].

Then in phase 2.2, comparison was made between the test lesion and the reference lesions created in the synthetic skin of the remaining objects (not excluded by comparison with reference lesions on modelling clay) based on general elements and dimensions. Indeed, phase 2.1 was intended to favour an initial sorting before phase 2.2 and to facilitate the reduction of possibilities to get closer to the object that created the lesion. Phase 2.2 allowed to eliminate more candidates to get closer to the right tool.

In cases of non-exclusion and when the reference lesions on synthetic skin showed acquired characteristics, comparison of this criterion with specific detailed characteristics on the test lesion was undertaken in phase 2.3. This was to check whether the features acquired in the test lesion were present in the reference lesions.

In phase 2.4, comparison between the test lesion and the reference lesions created in the synthetic skin and that were eliminated in phase 2.1 was undertaken. The aim of phase 2.4 was to assess whether the comparison made first in a softer (favourable) material could lead to false exclusions.

Finally, in phase 2.5, was undertaken the superimposition of the representations (2D, 3D, 3D colour) of the test lesion and reference lesions on the synthetic skin for those impressions whose dimensions led to exclusion or those that required justification to lead to non-exclusion (such as considering a bigger tolerance in difference of dimensions, or in difference of some shape parts). Superimposition is a method of comparison that allows to assess visually the degree of correspondence between a trace (the test lesion) and an impression (the reference lesion). The aim of phase 2.5 was to test whether the dimensions allow a correct conclusion of the comparison.

It is important to note that at the end of a comparison step, the following conclusions could be reached:

- Exclusion: the comparison revealed discordances in the general shape, or in the acquired characteristics without being able to explain them by the change in the surface of the blunt tool over a certain period.
- Non-exclusion: the comparison showed agreement and no unexplainable discordances that would tend to exclude.

At the end of the comparison procedure, after considering and discussing the results, a list was produced stating for each test lesion the blunt tools that could not be excluded as having produced them. The operator did not carry out an evaluation of the value of the association (quantification of the probative value), except for the fact of being able to exclude a tool. As the reference lesions were produced under known conditions, the confrontation of the results provided by the main operator with the form filled in by the volunteers (i.e. the tool that actually produced the test lesion) led to an evaluation of the overall method and to the formulation of paths for methodological improvements.

## 3 Results

### 3.1 Analysis

A total of 195 images – representing 65 lesions, documented with 3 different techniques – were processed in the Analysis phase. 64 were categorized in the high quality class, 69 in the medium quality one, and 62 were of low quality (Table 1).

**Table 1:** Total of the different “Quality of the representation” and “Conclusion” attributed to the 65 test lesions analysed by the combined three techniques.

Conclusion	Quality of the reprensation			
	High	Medium	Low	Total
Exploitable	63	33	5	*101*
Exploitable for exclusion only	1	36	40	*77*
Inexploitable	0	0	17	*17*
Total	*64*	*69*	*62*	*195*

In addition to assessing the quality, each representation was categorized according to its exploitability: 101 were considered as ‘exploitable’, 77 as ‘exploitable for exclusion only’ and 17 deemed ’unexploitable’ (Table 1). Despite the variation in quality among the images, the analysis phase led to the finding that the majority (178 of the 195 lesions representations) can be used for comparison, at least for exclusionary purposes, or even for initiating an identification procedure.

### 3.2 Comparison

43 out of 129 comparisons on modelling clay and 45 out of 129 on synthetic skin led to a non-exclusion.

In 14 cases, the comparison led to the conclusion of ‘exclusion’ of reference lesions on modelling clay, and to a non-exclusion with reference lesions on synthetic skin.

**Fig. 3:**
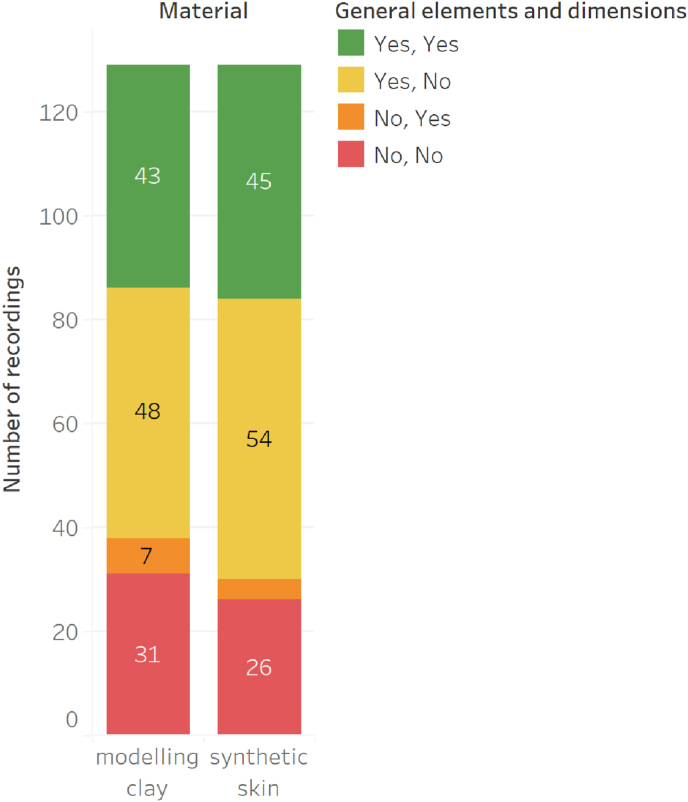
Concordance between the general elements and dimensions on each substrate.

The comparison process was based on two main criteria: the concordance of general elements and the matching of dimensions (Fig. 3).

In most comparisons, the general elements matched between the test and the reference lesions. Overall, more comparisons were positive on the synthetic skin than on the modelling clay. For many comparisons, the dimensions did not match between the test and the reference lesions. This trend was observed for both substrates in the same proportions (Fig. 3), suggesting that these discrepancies in dimensions were not influenced by the type of substrate. In most of these cases, these differences could be justified by arguments such as difference of elasticity between substrate, tolerance in dimension's measurement to consider or some limitations of the lesion not observable, leading to non-exclusions.

In a second step, we examined the acquired characteristics for all non-exclusions observed between test and reference lesions on synthetic skin (Phase 2.3 of the comparison). Majority of test lesions lacked acquired characteristics (n = 31), which made it impossible to perform comparison to the references. In few cases, acquired characteristics were recorded (confirmed by direct examination and excluded as artefact) but only some led to matches (n = 6 out of 14).

The reliability of the dimension (as criteria for comparison) was evaluated by superimposition of test and reference lesions represented on the synthetic skin. This was performed only on lesions whose dimensions led to exclusion or those that required justification to lead to non-exclusion (Table 2).

**Table 2:** Summary of conclusions obtained by contrasting the comparison on synthetic skin with the superimposition.

Conclusions	Total
Only one object retained and the same	20
Only one object retained and not the same	0
Only one object retained on synthetic skin and many by superimposition including the one on synthetic skin	8
Only one object retained by superimposition and many on synthetic skin including the one by superimposition	1
Many object retained in both situations	2
No object retained on synthetic skin	4
No object retained by superimposition	1

In most situations (20 on 36), the same single object has been retained. In some cases, no object was retained by comparison on synthetic skin, whereas there were some by superposition. By superimposition, more situations where several objects had not been excluded were encountered than in the comparison. A maximum of two to three objects were retained in each of these situations.

Looking at the detailed results of the superimposition vs the comparison on synthetic skin, the following findings were made:

- In 20 out of the 94 superimpositions carried out, the conclusion changed from exclusion to non-exclusion;
- In 6 cases out of 94, the conclusion changed from non-exclusion to exclusion.

### 3.3 Confrontation

**Fig. 4:**
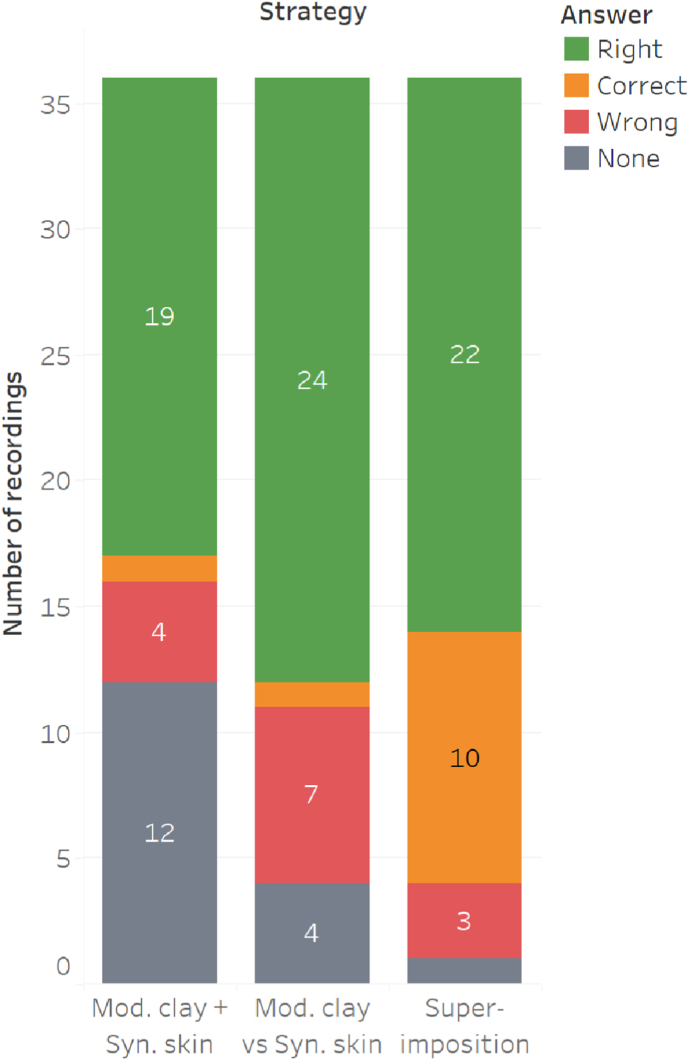
Accuracy of answers given according to the comparison strategy. In green are presented the answers where only one object was retained and the right one, in orange those where the object is present in the group of retained objects so considered as correct answer, in red the answers where the correct object was not retained and in black those where no object was retained. “Mod. clay” stands for “Modelling clay” and “Syn. skin” for “Synthetic skin".

At the end of the analysis and comparison procedures, the main investigator was informed of the answers (which object actually created which lesion). First, for the 12 test lesions compared, the answers given were compared with the solution (Fig. 4).

Our research focuses on the analysis phase, but it is important to consider the different comparison methods in order to verify whether the results obtained are attributable to the proposed analysis method or to certain comparison parameters. Indeed, the comparison phase should allow to verify the reliability and the usefulness of the developed analysis method, and it is important to differentiate the limits of each technique in order to highlight these points.

The two first comparison strategies (comparison on preliminary modelling clay then on synthetic skin (phases 2.1 and 2.2) or comparison only on synthetic skin (phase 2.4)), are very discriminating which leads to keeping only the “right object” or the “wrong one”. The comparison strategy based on the synthetic skin alone resulted in more right answers. The strategy using both substrates (first comparison strategy) allowed to exclude all objects in more cases than the second comparison strategy. These results raise the question of the usefulness of the modelling clay comparison, as it undermines the results obtained on the synthetic skin. Superimposition resulted in fewer errors and was less discriminative as it forced the exclusion of all objects in a single case compared to the other two strategies suggesting that a superimposition is sufficient, without a detailed analysis of the lesion.

To understand whether the entire traditional analysis-comparison or only certain points are unreliable, it is necessary to look in detail at the situations where a wrong answer was given by the first two comparison strategies and not by the superimposition. We focused our evaluation on cases where all objects were excluded. In all these situations, the general elements corresponded between test and reference lesions. For the cases where the wrong object was selected, the dimensions always had to be justified in order not to exclude except for one situation where this was not necessary. Thus, the problem is not the analysis process or the comparison in general, but the dimensions.

In about 35 % of the cases, the analysis method leads to situations of exclusion of the good object (either in favour of a bad one, or by keeping none), all detailed comparison techniques taken together (comparison on preliminary modelling clay or only on synthetic skin).

## 4 Discussion

The purpose of our research was to set up an experimental study to assess if the development ofan approach based on ACE-V could be beneficial for the exploitation of patterned injuries applying 3DSS; and then to lay the foundations for such a method. Our approach emphasises the analysis step, which should make it possible to clarify the criteria to be recorded on a lesion. These criteria should serve as a basis for

1. Determining what degree of association can be expected with an object suspected to have caused the lesion;
2. Guiding the comparison process.

The results obtained from these experiments can be used to develop a working method for this type of lesion.

This study is the first one to test if the use of ACE-V methodology could allow to analyse a patterned injury and then reduce the list of suspected objects that could have created this patterned injury (i.e. the lesion) on human skin. Previously, the methodology for selected potential suspected objects included only superimposition of the lesion's and the suspected object's representation, with no previous analysis. The main objective was to test the first step of the ACE-V methodology, which comprise analyzing the representation of the trace (in this case the lesion remaining on the skin produced by the suspected object) in order to determine if the trace is unexploitable, exploitable for exclusion only, or exploitable for further comparison. The results of the comparison phase were also used to verify the reliability of the analysis phase.

Furthermore, we carried out an internal test in which our two experts analysed a number of selected lesions, and a strong correlation in their conclusions was observed. Then, on the basis of the good degree of reproducibility obtained during this first step, we decided to continue the study of the other lesions with only one expert, as it was a preliminary study.

During the analysis phase, we were able to refine the degree of usability of each representation. Specifically, we differentiated between representations that could only be used to exclude objects as possible sources, and those that could be used to reach a more informed decision. For example, some representations allowed us to reduce the number of candidates to a limited set of objects, or even to a specific class of objects. This gradation of the anticipated conclusions at the end of the analysis phase allowed us to optimize our approach and, ultimately, to avoid the risk of overestimating the scope of subsequent comparisons.

The high rate of exclusion of the correct object due to inclusion of certain criteria demonstrates that this method of analysis in not totally reliable and has some limitations, mainly due to the use of dimensions. Differences in the conclusions of comparisons between the two materials could be due to the fact that the dimensions measured between the synthetic skin and the modelling clay can vary due to the different reaction of the substrate to the blow. Other results of the comparison phase indicate that the difference in substrate does not seem to influence the need for explanations. These results highlight the fact that the dimensions represent a less reliable criterion for comparisons. The measurement of dimensions is common in the analysis of traces in criminal science. It is reliable information relevant to the ACE-V process. However, in forensic medicine, the measurement of the dimensions of a lesion is not useable due to the particular nature of the skin. It is descriptive information of what is observed, but the results from our experiments suggest that it is not a suitable criterion for comparison purposes. Indeed, a difficulty in taking measurements on many lesions was noted, the edges being often unclear and the surface topography very variable. It was often difficult to define two precise points between which to measure the distance. Indeed, human skin is a soft tissue with particular reactions and has a high degree of elasticity. The skin sinks under the force of the blow and then frequently returns to its original position. If a slightly different angle is applied between several blows, differences in the final morphology can occur. Thus, measurements taken on one lesion can hardly be compared with those taken on another. Gaus et al. [39] illustrate this argument very well in their book. Although a sharp object is used in this example, the final reaction of the skin surface is similar to that struck by a blunt object. The only exception to this argument is in the skull region, where the skin is very close to the cranial cap, and the rigid structure of the bone under the skin means that the lesion is better fixed. The human skin simulation material used in this research also had a certain elasticity as well as a certain capacity to tear. The reaction of the material does not allow for a reliable evaluation of the dimensional measurements in the analysis and comparison. Thus, this criterion should only be used in the description of the lesion. The measurement of lesions should be done according to the different situations encountered (when a standard length and width cannot be taken). The dimensions of the features are also measured. Then, due to the particular nature of the skin and its substructures, and the weak (diffuse) nature of the patterned injuries (making precise and accurate measurement difficult), the measurement of dimensions is not a discriminating feature to be considered in the analysis phase.

The results of the comparison of **acquired characteristics** indicate that this element is not reliable to look for in this type of lesion and will not have informative potential for their exploitation. The type of lesions studied in this research is not very informative in terms of acquired characteristics. Indeed, few were observed, and they proved to be not very specific. Thus, this element is not of interest in the analysis of patterned injuries. The information content for individualisation is therefore limited. The evidential value of this type of trace is weakened in comparison with shoe marks or tool marks. The reason for this is inherent in the special properties of the skin as a support. The highly questionable use of dimensions and the low occurrence of acquired characteristics inevitably mean that the comparison procedure cannot lead to conclusive findings. This must be considered in the conclusions that are reached by this type of comparison. The specific methodology developed for this particular type of trace must therefore be revised.

Two different approaches inspired from the ACE-V approach were tested for the comparison phase in order to verify the relevance of the use of the reference lesions in modelling clay in the comparison. It was thus observed that during the comparison on modelling clay and then on synthetic skin, less “correct answers” (the right object was not excluded) were obtained than during the comparison directly on synthetic skin. It led to the exclusion of all the objects in most? cases, but it allowed to make less errors. The superimposition allowed less error and total exclusion than the other two methods but was less selective and discriminating. This could be due to the fact that it is not based on numerical measurements but on an overall visual assessment, allowing for a greater tolerance. Thus, it seems that proceeding with a first comparison phase on modelling clay removes candidates that could be matched. Therefore, these findings will allow a different exploitation of the reference lesions in the softer material during the comparison as well as a revaluation of the place given in the superimposition in the process. As the comparison is intended to verify the analysis protocol, it must also have as little bias as possible. Otherwise, this could make it more difficult to evaluate the analysis protocol.

In this study, two models were used to mimic human skin (modelling clay and synthetic skin) during comparison in combination with various objects. The results of this study allowed establishing an analytical protocol that can be used to categorize the trace and if it is exploitability, then narrow down the category of the suspected objects. Indeed, in most cases, the conclusion of the analysis was consistent with the comparison results and allowed to identify the right object or to keep it as having possibly produced the test lesion among 2 to 3 objects. This established analytical protocol highlights the information conveyed by the trace. Several criteria and their usefulness and relevance were evaluated, namely clarity of the lesion, its general pattern and dimensions, observed features and the quality of each one.

In this study, photography and surface scanners were chosen as recording techniques for the lesions based on our routine work in our institute and the advantages of these techniques. Photogrammetry was not chosen based on the following arguments. We agree that improvement have been recently made with photogrammetry, that it is nowadays routinely used in some medico-legal institutions [40]. However, this technique is still taking more time than 3D surface scanner or photography, giving lower-quality volume rendering than structured-light 3D scanners with no real-time control of three-dimensional data. Moreover, it needs more postprocessing and skills to see the final result. That's why we didn't include it in our first study. But further research applying photogrammetry would be welcome to complement this study.

The main limitations of this study are that the properties of skin's models do not fully resemble real skin. The choice of the simulation material was made by internal tests comparing materials found in the literature and following up with colleagues in the forensic field. Blows were applied to the materials and 2D and 3D acquisitions were made and compared. The material selected, synthetic skin commonly used by novice tattooists to learn how to tattoo, was the one that best met the criteria required for our study: skin-like reaction in terms of plasticity and elasticity, availability, cost, ease of use, stability over time and storage. As there is some difference in synthetic skin and real skin reaction to a blow, a higher exclusion rate would be expected in real situations. Both materials don't have exactly the same elasticity, and the subcutaneous tissues also impact its reaction. Ballistic soap used here also have an influence on synthetic skin reaction. Indeed, no experimental material tends to react exactly like this particular substrate that human skin and subcutaneous tissues are. Also, the comparison and confrontation have been studied for only 12 test lesions, all of which can be exploitable with at least one of the techniques and are of good or average quality. Our experimental results tend to show what is achievable in the best case.

Based on our experimentations and all these observations, we will refine our analysis protocol in order to improve its relevance and usefulness so as to test its applicability and validity in other experimental research and also on real cases. An improved analysis form was produced considering: a) clarity and quality of the lesion; b) general pattern and its quality; c) specific characteristics and their dimensions and quality. This information allows to dress a conclusion of the analysis: Unexploitable, Exploitable for exclusion only, Exploitable. General dimensions (length and width) of the lesion are also measured but won't be considered for the conclusion. The change is also in the way of evaluating the quality of the lesion and its informative value, considering the particularity of the medium. Before this study, the impact of the elasticity of the support, the difficulty of printing the pattern and the shape of the object were underestimated, giving very optimistic conclusions about the usability of the lesion. With knowledge of these aspects in the new analysis, a more realistic estimate can be made avoiding time consuming analyses which cannot lead to a clear conclusion. Then this new analysis protocol will be tested through the current experimentation on real lesions in order to fully verify its applicability and benefits in real cases.

## 5 Conclusion

The purpose of our experimental study was to assess if the development of an approach based on ACE-V could be beneficial for the exploitation of patterned injuries applying 3DSS. The results obtained partly demonstrate its potential for this use. Our results tend to show what is achievable in the best case (situation in which the trace is of the highest quality and is as informative as possible in order to provide the most convincing conclusion) using analysis protocol and also highlight some limitations of our approach. This research was conducted on lesions created on simulation material which obviously differed from human skin. However, the results obtained under experimental conditions are very encouraging and have allowed an initial evaluation of the analysis protocol and its improvement. It is thus planned to test this improved analysis protocol in new experimentations and on real lesions to fully verify its applicability and benefits in real cases. This study is a first step, proposing a promising approach that will be validated in a second stage. It is a proof of concept that requires further investigation, and we encourage further research in this area.

## CRediT authorship contribution statement

**Stella Fahrni:** Writing – original draft, Visualization, Methodology, Investigation, Conceptualization. **Olivier Delémont:** Writing – review & editing, Supervision, Project administration, Conceptualization. **Silke Grabherr:** Writing – review & editing, Supervision, Project administration, Conceptualization.

## Declaration of competing interest

The authors declare that they have no known competing financial interests or personal relationships that could have appeared to influence the work reported in this paper.
