# Supplementary material for: Forensic exploitation of patterned injuries: Promoting structured analysis as an early assessment for comparison process
Source: Forensic Sci Int Synerg. 2024 Apr 23;8:100469. doi: 10.1016/j.fsisyn.2024.100469 (PMC11061693; doi:10.1016/j.fsisyn.2024.100469)
Supplement: Multimedia component 1 [file mmc1.pdf]

L030

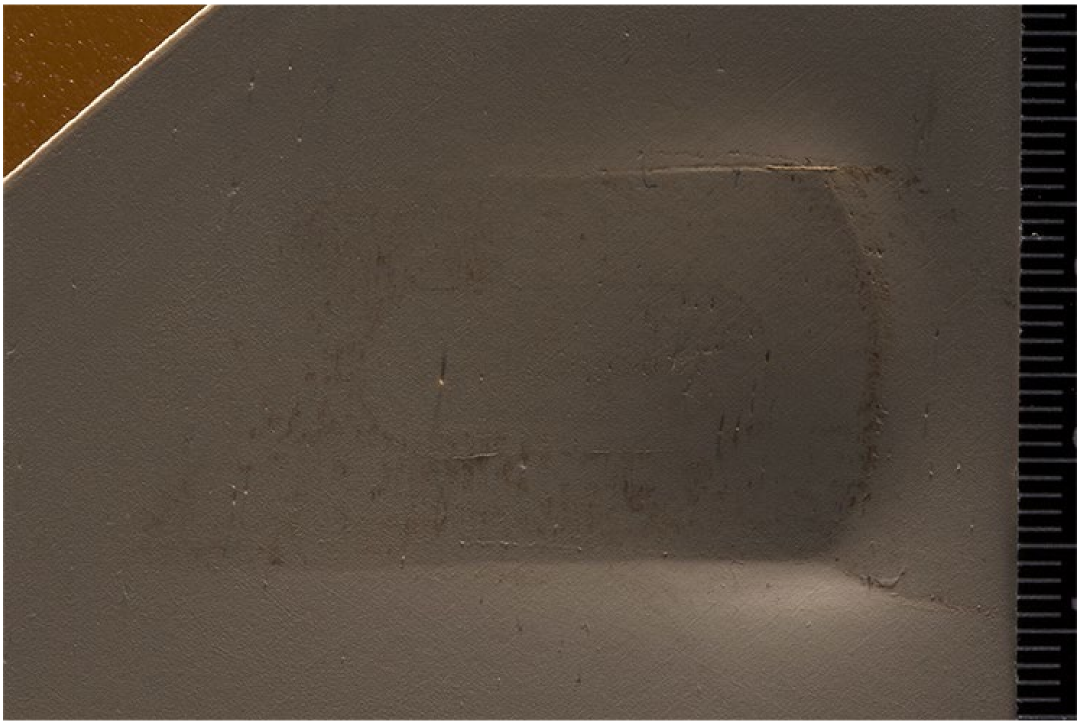

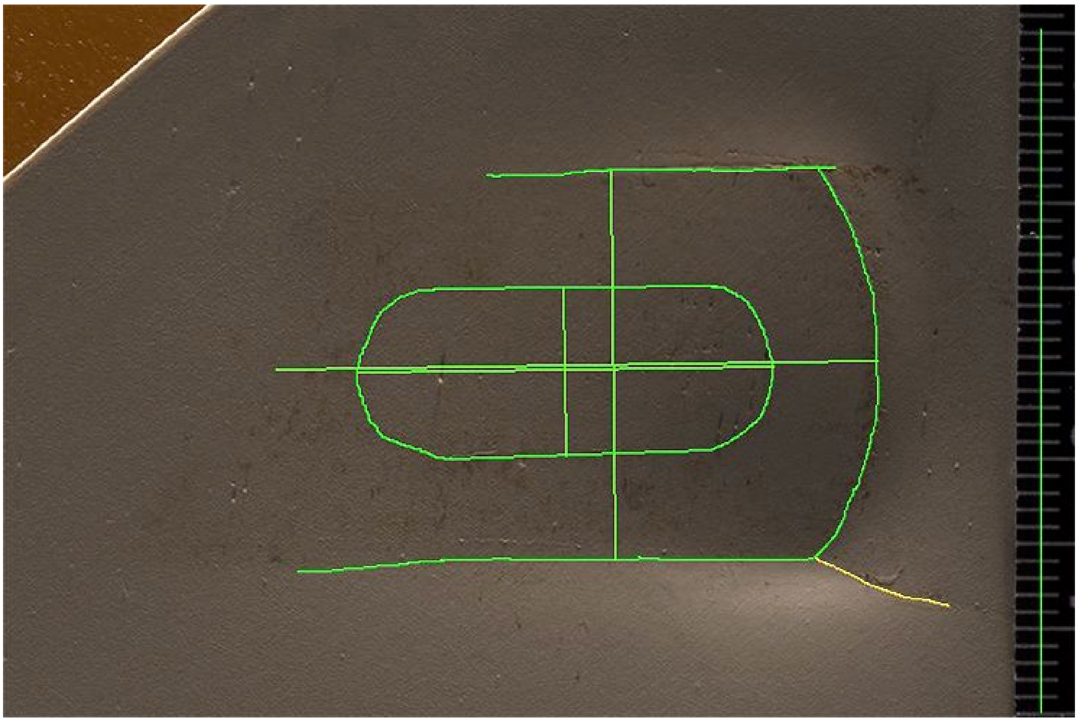

Participant : 1 Date : 20.03.2018

Trace analysed : L030

Trace origin : Tool

Recording technique : 2D

Substrat : Synthetic skin

Type of lesion : Contusion

Material : Dust

Type of trace : Molded

Represented area :

Clarity and overall quality of the trace :

The trace is moderately marked. There is some visible detail. There does not appear to be any distortion, double apposition or slippage. There is some dirt on the trace, but this seems to help to see boundaries rather than obscure legibility. Overall, the trace is of average quality.

Dimensions : 3.50 (G) x 2.29 (G) cm. Minimum dimensions expected

**Observed features** :	G/Y/R
The trace is rectangular with a rounded right-hand edge. The left side edge is not visible. The bottom and right edges are clearly visible in the dust, as is part of the top edge.	G<br>G
The trace appears to be slightly sunken.	
In the center of the trace, there is an oval-shaped pattern that is also drawn in the dust: 2.43 x 0.98 cm (G)	G
A line can be seen running outwards from the bottom right-hand corner.	Y
In the top right-hand corner, the horizontal line of the border is also more pronounced and extends a little beyond the outline.	G

Conclusion : Exploitable

Argumentation :

The trace is moderately marked. There is some visible detail. There are no signs of distortion. The trace is rectangle-shaped with a rounded right edge. The edges of the trace are visible except on the left end and part of the upper part. The trace is slightly sunken. An oval-shaped pattern is visible in the center of the trace. The contours and pattern are highlighted by the arrangement of the dust. Overall, the trace is of medium quality.
